# Middle trapezius transfer for treatment of irreparable supraspinatus tendon tears- anatomical feasibility study

**DOI:** 10.1186/s40634-021-00326-1

**Published:** 2021-01-23

**Authors:** Philipp Moroder, Doruk Akgün, Lucca Lacheta, Kathi Thiele, Marvin Minkus, Nina Maziak, Thilo Khakzad, Christian Festbaum, Katja Rüttershoff, Sophia Ellermann, Torsten Weiss, Thomas Jöns, Victor Danzinger

**Affiliations:** 1 Department for Shoulder and Elbow Surgery, Center for Musculoskeletal Surgery, Campus Virchow, Charité -Universitaetsmedizin Berlin, Augustenburgerplatz 1, 13353 Berlin, Germany; 2 Department for Anatomy, Institute for Functional Anatomy, Center for Surgical-anatomical Training, Charité -Universitaetsmedizin Berlin, Berlin, Germany

**Keywords:** Irreparable rotator cuff tendon tear, Irreparable supraspinatus tendon tear, Tendon transfer, Middle trapezius transfer, Anatomical study

## Abstract

**Purpose:**

The purpose of this study was to investigate the anatomical feasibility of a middle trapezius transfer below the acromion for treatment of irreparable supraspinatus tendon tears.

**Methods:**

This study involved 20 human cadaveric shoulders in 10 full-body specimens. One shoulder in each specimen was dissected and assessed for muscle and tendon extent, force vectors, and distance to the neurovascular structures. The opposite shoulder was used to evaluate the surgical feasibility of the middle trapezius transfer via limited skin incisions along with an assessment of range of motion and risk of neurovascular injury following transfer.

**Results:**

The harvested acromial insertion of the middle trapezius tendon showed an average muscle length of 11.7 ± 3.0 cm, tendon length of 2.7 ± 0.9 cm, footprint length of 4.3 ± 0.7 cm and footprint width of 1.4 ± 0.5 cm. The average angle between the non-transferred middle trapezius transfer and the supraspinatus was 33 ± 10° in the transversal plane and 34 ± 14° in the coronal plane. The mean distance from the acromion to the neurovascular bundle was 6.3 ± 1.3 cm (minimum: 4.0 cm). During surgical simulation there was sufficient excursion of the MTT without limitation of range of motion in a retracted scapular position but not in a protracted position. No injuries to the neurovascular structures were noted.

**Conclusion:**

Transfer of the acromial portion of the middle trapezius for replacement of an irreparable supraspinatus seems to be feasible in terms of size, vector, excursion, mobility and safety. However, some concern regarding sufficiency of transfer excursion remains as scapula protraction can increase the pathway length of the transfer.

**Level of evidence:**

Basic Science Study/Anatomical Study

## Introduction

Irreparable supraspinatus tendon tears (ISTT) with associated pain and loss of function are difficult to treat especially in a younger and high-demanding patient population. Reverse total shoulder arthroplasty may not be the primary option for these patients due to limited longevity and high risk for complications [7, 16]. Therefore, different joint preserving procedures to reduce pain and improve function have been proposed including partial repair, superior capsular reconstruction, subacromial spacers, and interposition tendon grafting [6, 24, 32, 33]. Despite early clinical benefits, partial repair does not restore the former full coverage of the humeral head by the cuff and interposition grafting depends on the still available rotator cuff muscle tissue. Superior capsular reconstruction and subacromial spacers emerged with similar promising short-term results [23] but do not provide a dynamic biomechanical component.

Tendon transfers are common salvage procedures for irreparable rotator cuff tendon tears (IRCTT) featuring a dynamic biomechanical component and independence from residual muscle and tendon tissue of the torn musculotendinous unit. While several replacement options have been described for anterosuperior and posterosuperior IRCTTs [9, 10, 12, 15, 18, 19, 27, 28, 36], currently, no tendon transfer option other than the deltoid-flap procedure [2, 34] is available for replacing a ISTT.

While trapezius transfers have been previously used for paralytic patients due to brachial plexus palsies and deltoid muscle inactivity [1, 3–5, 13, 20, 22, 25, 29, 30], a transfer of the middle trapezius to treat patients with ISTT has so far not been described. Therefore, the purpose of this study was to investigate the anatomical feasibility of a middle trapezius transfer below the acromion to substitute the supraspinatus.

## Methods

To evaluate the feasibility of the proposed middle trapezius transfer (MTT), an anatomic study was performed including 20 cadaveric shoulders in 10 fresh-frozen full-body specimens. One shoulder of each specimen was used to identify and assess the anatomical characteristics of the trapezius muscle and tendon by means of a complete anatomical dissection and the other side to evaluate the surgical feasibility of the MTT when performed via limited skin incisions.

All specimens were descended from the body-donor program of the institutional center of anatomy. Specimens included 6 female and 4 male donors with an average age at the time of death of 81 ± 7 years. Only specimen with intact rotator cuff and no evidence of advanced osteoarthritis were employed for this study.

Institutional ethical committee approval was obtained prior to the beginning of this study.

### Anatomical assessment

With the specimens in prone position, the C7 spinous process was used as anatomic landmark to perform a wide T-shaped incision to the acromion and in line with the spine. The skin and the subcutaneous tissue were carefully removed to reveal the origin and insertion of the trapezius muscle. The trapezius muscle was separated into upper, middle and lower trapezius based on the definition by Omid et al. who use the triangular aponeurosis as reference to identify the middle trapezius [26]. The rostral and caudal extend of the separate muscle portions was noted. Muscle fibers of the upper, middle and lower trapezius were followed towards lateral and their insertions were analyzed by noting the anatomical landmarks. Furthermore, the length of the muscular and tendinous portions as well as the width at origin, insertion, and the myotendinous junction of the three parts of the trapezius were quantified using a tape measure. Additionally, the length of the clavicle, acromion, and scapular spine was assessed.

**Fig. 1:**
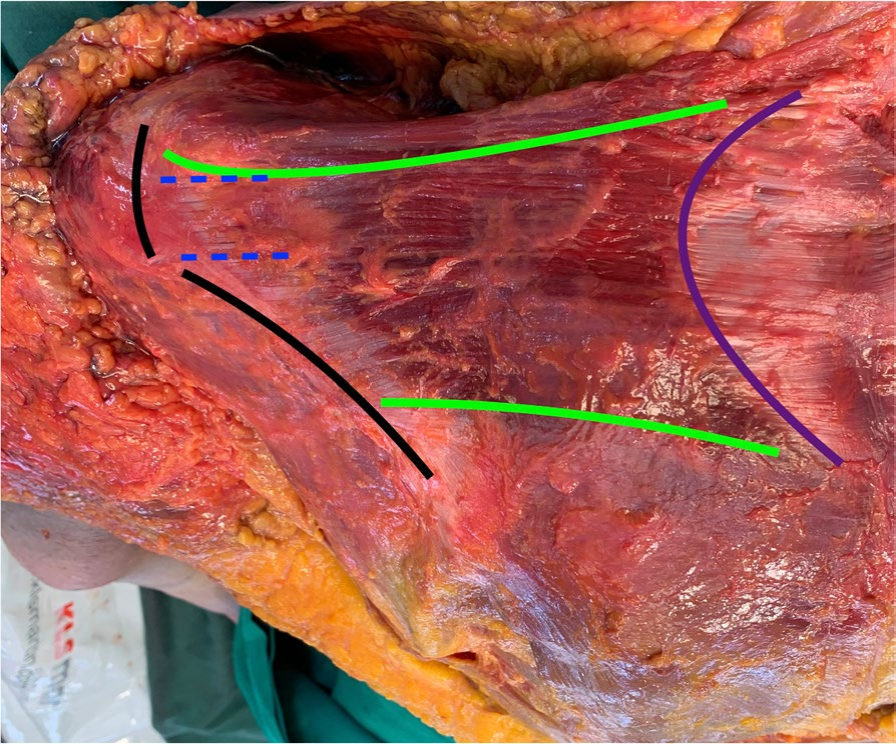
Posterior view on the middle trapezius muscle with the specimen in prone position. The middle trapezius (between green lines) originates from the aponeurosis (purple line) and extends laterally until inserting into the lateral two thirds of the scapula spine and the acromion (black lines). It can be observed that the upper part of the middle trapezius curves posteriorly around the lateral clavicle and AC joint. The blue dashed lines mark the proposed harvesting site for a middle trapezius transfer (MTT) for treatment of irreparable supraspinatus tendon tears

After visual inspection regarding force vector, size, as well as proximity to the greater tuberosity, the ideal portion of the middle trapezius transfer (MTT) to replace ISTTs was identified to be the part which inserts medially at the acromion just posterior to the acromioclavicular (AC) joint and anterior to the spina scapulae (Fig. 1). After subperiosteal detachment from the acromion, the tendon thickness of the MTT was measured at the anterior, middle and posterior end and the tendon footprint length and width were determined. Next, the remaining trapezius muscle fibers were reflected off their scapular and clavicle insertion to expose the spinal accessory nerve as well as the superficial transverse cervical artery on the ventral muscle belly. The neurovascular route was then marked from superficial using several 18 gouge needles and the minimum distance from the acromial footprint to the neurovascular pathway was measured. The angle between the force vectors of the MTT and the underlying supraspinatus were determined in the transversal and coronal plane using a goniometer. An osteotomy between the spine of the scapula and the acromion was performed to expose the humeral head. The supraspinatus was detached from its footprint and the tendon thickness was measured at the anterior, middle and posterior end. The length and width of the footprint was noted. After performing the transfer, the shortest distance from the former acromial footprint to the neurovascular pathway as well as the vector angles were then re-assessed.

### Surgical feasibility

The second step investigated the surgical feasibility of the MTT with regard to the aforementioned anatomical structures. The clavicle, AC joint, acromion, and scapular spine were palpated and a 5 cm incision was performed parallel to the anterior border of the spino-acromial junction. The acromial insertion of the middle trapezius was identified and released subperiosteally. According to the safe zone established in the anatomical assessment, a limited longitudinal split of the middle trapezius of 3 cm was performed and the released tendinous portion was armed with two #2 non-absorbable sutures using Krackow stitches. Next, a deltoid split approach was completed and the supraspinatus tendon was excised. The armed MTT was then transferred underneath the acromion and attached to the former supraspinatus footprint using transosseous tunnels. After attachment, potential blockage of shoulder motion by the transfer was assessed. Finally, the trapezius muscle belly was dissected to expose and confirm the intactness of the neurovascular bundle.

## Results

In most specimens the aponeurosis which defines the origin of the middle trapezius extended from the spinous process C5 (range C5-C7) to T3 (range T2-T5). While the upper trapezius origin could be traced to the external occipital protuberance in many cases, the most frequent caudal origin of the lower trapezius was the spinous process T10 (range T9-T12). Regarding its insertion, the upper trapezius attached to the lateral third of the clavicle in all cases. The middle trapezius insertion always included the entire medial edge of the acromion and approximately the lateral two thirds of the spine of the scapula. In three cases the middle trapezius insertion extended to the lateral clavicle as well. The lower trapezius involved the medial third of the spine of the scapula in all cases (Fig. 1).

The middle trapezius width was 11.4 ± 2.5 cm at origin, 8.5 ± 2.0 cm at the myotendinous junction, and 10.6 ± 3.0 at the insertion. The average middle trapezius muscle length was 11.7 ± 3.0 cm and the average middle trapezius tendon length was 2.7 ± 0.9 cm.

**Fig. 2:**
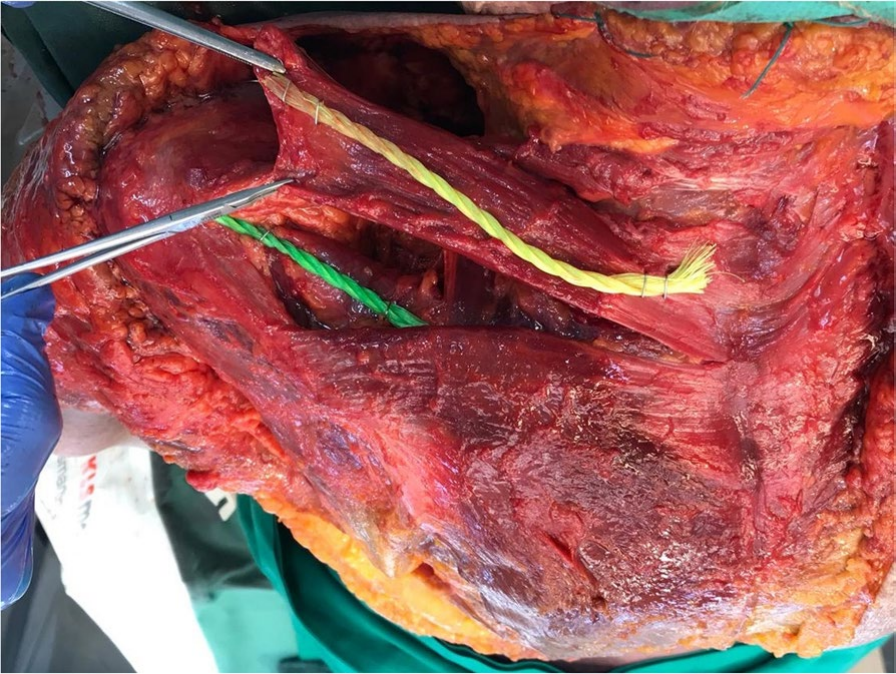
Axial view of a completely dissected and displaced middle trapezius transfer (yellow cord) which reveals the underlying supraspinatus (green cord) in close proximity and with a similar vector in the transversal plane

The footprint of the MTT had an average length of 4.3 ± 0.7 cm and a width of 1.4 ± 0.5 cm. The average thickness of the tendon was 0.4 ± 0.2 cm anteriorly, 0.5 ± 0.3 in the middle, and 0.5 ± 0.5 posteriorly. In comparison, the supraspinatus showed a mean footprint length of 2.6 ± 0.5 cm and width of 1.5 ± 0.7 cm as well as a tendon thickness of 0.4 ± 0.1 cm anteriorly, 0.4 ± 0.1 in the middle, and 0.5 ± 0.2 posteriorly. The average angle between the non-transferred MTT and the supraspinatus was 33 ± 10° in the transversal plane and 34 ± 14° in the coronal plane and after transfer 30 ± 13° in the transversal plane and 24 ± 9° in the coronal plane (Fig. 2).

**Fig. 3:**
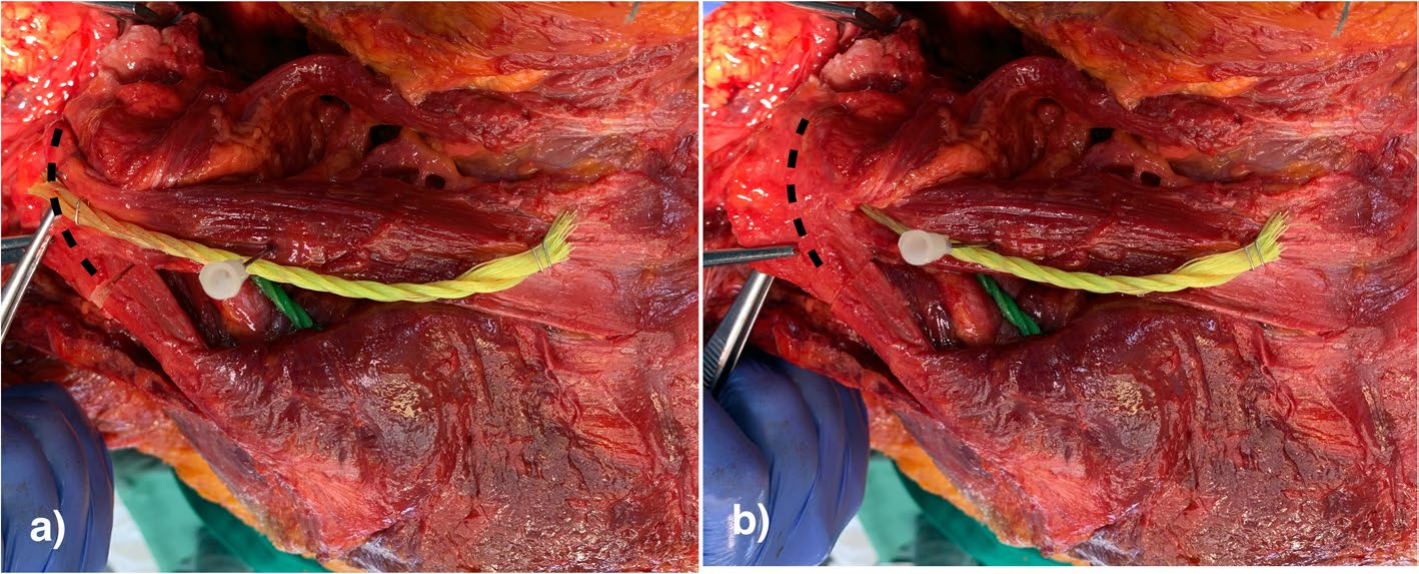
Axial view of a completely dissected middle trapezius transfer (yellow cord) after harvesting (**a**) and after transfer (**b**) below the acromion (dashed black line). The needle marks the furthest lateral position of the accessory nerve indicating the limits for mobilization of the middle trapezius transfer

The mean distance from the inner acromion edge to the neurovascular bundle was 6.3 ± 1.3 cm with a minimum of 4.0 cm. After transfer the mean distance from the inner acromion edge to the neurovascular bundle was 4.1 ± 1.2 cm with a minimum of 2.5 cm indicating an average lateral displacement of the accessory nerve of 2.1 ± 1.0 cm (Fig. 3).

The surgical simulation of the middle trapezius transfer did not reveal any injury to the neurovascular bundle. After the release within the safe zone, the transfer had sufficient length to reach the footprint on the greater tuberosity in all cases with the scapula manually retracted into an approximate position which would be expected in a standing position. When the scapula was left in protraction as caused by the prone position of the specimens with the thorax resting on a wooden block, the footprint could usually not be reached. After transfer no blockage of motion by the transfer when moving the arm of the specimen in prone position but with retracted scapula was observed.

## Discussion

Several tendon transfer options for irreparable rotator cuff tendon tears have been proposed [9, 10, 12, 15, 18, 19, 27, 28, 36]. Common transfer options include Pectoralis major and Latissimus dorsi for anterosuperior IRCTT and Latissimus dorsi and Lower trapezius for posterosuperior IRCTT. Even though it is the most frequently encountered pathology no tendon transfer options other than the deltoid flap procedure have been described so far for ISTTs. The goal of this study was to provide data on the anatomical feasibility of a partial transfer of the middle trapezius underneath the acromion as salvage option to treat ISTTs.

From an anatomical standpoint the trapezius muscle has a singular innervation by the accessory nerve (cranial nerve XI) and can be separated into three parts based on their origin above, at, or below the aponeurosis [26]. According to the study observations, the origins of the three parts of the trapezius as well as the extent of the aponeurosis along with its quite common variability were similar to a previous report [26]. When tracing the muscle fibers towards lateral, the upper trapezius inserts on the lateral clavicle, the middle trapezius mostly on the acromion and the lateral two thirds of the scapula spine, and the lower trapezius on the medial third of the scapula spine. Therefore, the acromial insertion can be attributed to the middle trapezius instead of the upper trapezius as stated contrary [17].

**Fig. 4:**
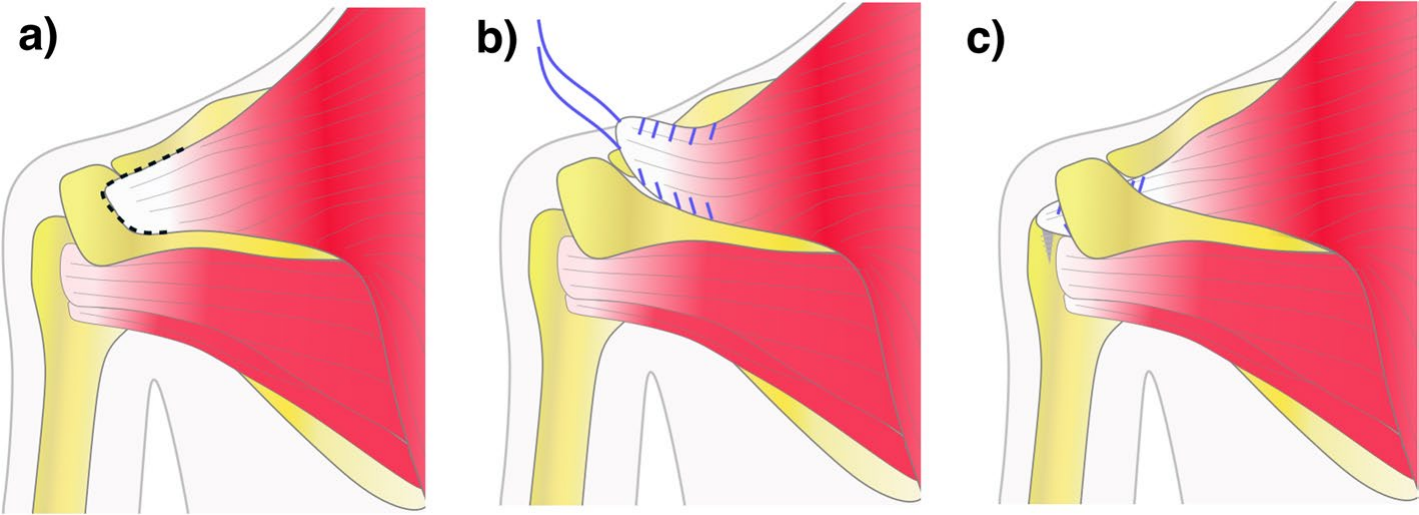
Schematic drawing of the middle trapezius transfer (MTT) for treatment of irreparable supraspinatus tendon tears. The figure shows **a** the native insertion of the acromial portion of the middle trapezius, **b** the subperiosteal detachment, and **c** the transposition through the subacromial outlet and re-attachment to the former footprint of the supraspinatus

The trapezius has been highlighted as a tendon transfer option for IRCTTs previously. While the transfer of the upper trapezius instead of the pectoralis major transfer to replace an irreparable subscapularis tendon has rendered unsatisfactory outcomes [14], the transfer of the lower trapezius instead of the latissimus dorsi transfer to replace an irreparable infraspinatus tendon showed promising early results [11]. In addition, trapezius transfers have been performed since many years in patients with brachial plexus palsies and deltoid muscle insufficiencies in order to counteract abduction paralysis [1, 3–5, 13, 20, 22, 25, 29–31]. Early descriptions date even back to Hoffa in 1891, Lewis in 1910 and Lange in 1911 [8]. The described techniques mostly involve some form of osteotomy of the spine of the scapula, acromion, or clavicle with detachment of the deltoid, transposition of the acromion to the humerus, as well as reinsertion of the deltoid on the trapezius. The reported improvements in abduction seem minor at first, however, still can be considered quite impressive giving the adverse initial situation with true muscle paralysis including the deltoid muscle [1, 3–5, 13, 20, 22, 25, 29, 30]. The initial situation in patients with ISTTs usually involves pain as well as loss of strength and limited motion [35] but typically shows a still functioning deltoid muscle. Therefore, the rationale behind the introduced middle trapezius transfer, is to transpose the acromial portion of the middle trapezius through the subacromial outlet and re-attach it to the former footprint of the supraspinatus while keeping the deltoid and the acromion intact (Fig. 4). If successful, this transfer provides a viable replacement to the superior aspect of the rotator cuff, that mimics its characteristics in terms of tendon dimension and force vector according to our study results. This combination of static spacing and dynamic joint centering effect might combine the benefits of both, static concepts seen in subacromial spacers or superior capsular reconstruction and dynamic concepts like partial rotator cuff repairs and interposition grafting [6, 24, 32, 33].

With the scapulae in a retracted position, the excursion of the musculotendinous unit seems sufficient without stressing the neurovascular supply and the transferred tendon does not appear to block motion in any direction due to excessive tension or notching. However, scapula protraction which increases the pathway length between origin and new insertion of the transfer at the greater tuberosity remains a concern. Therefore, the necessity for graft interposition in order to lengthen the transfer in potential clinical application in some cases cannot be excluded. This necessity of graft interposition could potentially be dynamically evaluated intraoperatively. In any case an extensive subperiosteal detachment of the tendon stump from the surface of the acromion seems to be of benefit in order to increase transfer length.

Form a surgical perspective, the MTT can be achieved via two incisions, one for the harvesting step, one for the fixation to the greater tuberosity, with the latter having potential to be performed arthroscopically. There is minimal risk for harming neurovascular structures during harvesting, transposition, and re-fixation of the tendon transfer due to the sufficient distance between tendon insertion and pedicle and limited lateral displacement of the nerve during transfer. However, separation of the harvested musculotendinous unit from the remainder of the trapezius should only be performed within the safe zones in order to protect the accessory nerve. When the harvesting is performed accurately, the loss of original function of the trapezius should be limited to a minimum as only a small portion of the entire muscle is transferred and the vector of pull of the transferred tissue remains similar. The size of the transfer can be increased by harvesting not only the acromial insertion but also a part of the spinal insertion in order to cover larger rotator cuff defects, however the further posterior the harvest site is extended the shorter that part of transfer becomes.

Due to the proximity of the middle trapezius that curves behind the AC joint, a critical step is the tendon harvest with potential risk of injury of the posterior AC joint capsule. A further possible pitfall is the rather short tendon stump of the MTT that warrants subperiosteal detachment from the acromion in order to provide sufficient length and tendon tissue to withstand suture rip-through. When armed with Krackow stitches sufficient suture stability was achieved in all specimens despite moderate tissue quality in these elderly specimens.

### Limitations

First, this anatomical study could not determine the activation pattern of the trapezius muscle which is innervated by the accessory nerve in contrast to the supraspinatus muscle, which is innervated by the suprascapular nerve. Nevertheless, the middle trapezius muscle activates synergistically to the supraspinatus muscle during arm elevation [21, 37] which provides a favorable base for the patients’ adjustment to the muscle transfer.

Second, the force production of the trapezius muscle was not assessed in this study. However, Herzberg et al. showed that the portion of the trapezius that inserts at the acromion reaches approximately two thirds of the force production potential of the supraspinatus muscle [17].

Third, the use of fresh frozen cadavers improves the comparability of the measurement parameters to the in-vivo situation, however, differences regarding tendon excursion, size of the musculotendinous unit, and range of motion are likely especially considering the advanced age of the specimens. Fourth, the comparison of the vectors between the MTT and the supraspinatus is affected by scapula position due to the difference of origin of the two muscles. Nonetheless, this study has shown that the vector of the MTT itself did not change relevantly in both planes after transfer.

## Conclusion

The results of this study confirm the anatomical feasibility of the transfer of the acromial portion of the middle trapezius for the treatment of irreparable supraspinatus tendon tears. Size, vector, excursion, and mobility of the transfer seem to be suitable to replace an irreparable supraspinatus. However, some concern regarding sufficiency of transfer excursion remains as scapula protraction increases the pathway of the transfer. The surgical risk of harming neurovascular structures while performing the transfer seems to be minimal.

## Data Availability

Data can be made available upon request.

## Abbreviations

ISTT: Irreparable supraspinatus tendon tears
IRCTT: Irreparable rotator cuff tendon tears
MTT: Middle trapezius transfer
AC joint: Acromioclavicular joint
